# An intergenerational reading of climate change-health concern nexus: a qualitative study of the Millennials’ and Gen Z participants’ perceptions

**DOI:** 10.1186/s12889-023-15353-z

**Published:** 2023-03-13

**Authors:** Ruxandra Malina Petrescu-Mag, Dacinia Crina Petrescu, Adrian Ivan, Ancuta Tenter

**Affiliations:** 1 Department of Environmental Science, Faculty of Environmental Science and Engineering, Babes-Bolyai University, Cluj-Napoca, Romania; 2 Department of Economy and Rural Development, Faculty of Gembloux Agro-Bio Tech, University of Liège, Liège, Belgium; 3 Doctoral School “International Relations and Security Studies”, Babes-Bolyai University, Cluj-Napoca, Romania; 4 Department of Hospitality Services, Faculty of Business, Babes-Bolyai University, 7 Horea Street, Cluj-Napoca, 400174 Romania; 5 Department of International Studies and Contemporary History, Faculty of History and Philosophy, Babes-Bolyai University, Cluj-Napoca, Romania; 6 Applied Environmental Research Centre, Faculty of Environmental Science and Engineering, Babes-Bolyai University, Cluj-Napoca, Romania

**Keywords:** Climate change anxiety, Health, Attitude, Barriers, Generations

## Abstract

**Background:**

The study of climate change through a generational lens is meaningful when one considers the distinct attitudes, behaviors, values, and motivations of each generation. Individuals born between 1980 and 1999, referred to as the Millennial Generation (Millennials) and individuals born up to five years before or after 2000, referred to as Generation Z (Gen Z), may differ widely in their views, values, attitudes, and behaviors. This may lead to conflicts between these two cohorts. As Gen Z enters the labor market, their first-level supervisors will be, in many cases, the Millennials, who may view the topic of climate change-health concern nexus very differently than their Gen Z subordinates. Considering the perspectives of each generation may offer insights on how to engage them to act in an environmentally responsible way to counteract climate change effects.

**Objective:**

The study reveals similarities and differences in how Millennials and Gen Z perceive the climate change-health concern nexus, which illuminates the understanding of the potential generational conflicts and the critical points where intervention is needed.

**Method:**

Interview data from 41 participants were analyzed via thematic analysis using the *Quirkos* software program. Reporting is in accordance with the *COREQ* guidelines.

**Results:**

The interview questions elicited responses related to five dimensions: (i) Views of individual and community health; (ii) Knowledge around climate change; (iii) Perceived health impact; (iv) Attitudes towards climate change; (v) Behaviors related to climate change. The findings revealed a set of commonalities and differences in understanding the climate change-health concern nexus between the participants representative of each of the generations examined. One main result is that while most interviewees perceived changes in summer and winter temperatures, they failed to articulate how climate change affected their health.

**Conclusion:**

Thematic analysis revealed that the commonalities of views outweigh the differences between the two generations. A relevant remark is that participants can be described rather as “observers” than “players” since they do not tend to see themselves (through their behavior and their contribution) as active participants in the goal to fight climate change. Consequently, both generations undergo what Stephen Gardiner [1] called “intergenerational buck-passing.”

**Supplementary Information:**

The online version contains supplementary material available at 10.1186/s12889-023-15353-z.

## Background

The study of climate change through a generational lens is meaningful when one considers the distinct attitudes, behaviors, values, and motivations of each generation. Therefore, we can generate climate change strategies tailored to fit people’s characteristics and values. Even though we are different at the individual level, considering the perspectives of each generation may offer insights on how to act more environmentally friendly.

Many people often perceive climate change as a distant phenomenon – temporally, spatially, and socially –from their everyday experiences [2]. That is why they often turn to their values, motivations, and personal experience to provide cues about climate change [3]. Research [4, 5] shows that the values of a generation are influenced by their prior social life experiences during the historical period in which they were born and raised. Since climate change discourse and action have also been marked by the intergroup dimension [6], an intergenerational reading of climate change is important for better managing climate change. Intergroup behavior may refer to differences and similarities between groups (e.g., generations, vulnerable groups like ethnic, women, and religious groups) regarding how people perceive, think, feel about and act towards (in our case) climate change and relate to people in other groups [7].

### Research objective and exploratory question

While scientific literature has mainly looked at generational differences in a variety of work and vocational contexts [e.g., 6–10], there is little research focused on generations’ perspectives of similarities and differences in climate change issues [6, 11–14].

To fill in this knowledge gap, the main objective of this study is to provide an intergenerational reading of similarities and differences in the climate change-health nexus that can illuminate the understanding of the potential generational conflicts and the critical points where intervention is needed. Accordingly, the following exploratory question (EQ) is introduced to respond to this objective: “How do the two selected generations understand and interpret the climate change-health nexus?”

### Theoretical framework

#### Climate change-human health nexus

The current and expected economic, social, environmental, and political challenges posed by climate change are evidenced by a rich scientific literature, transforming climate change into what Butler [15] called the “most existential problem of the 21st century.” Climatic conditions influence the ecosystem’s function and quality, the quality and quantity of food production, and therefore, the critical relations between climate, society, and the food system must be acknowledged [16, 17]. Climate change impacts on health are influenced by economic and social conditions and other components of the natural and human systems [18]. Climate change, considered “the single biggest health threat facing humanity” [19], impacts health in countless ways. For example, biological sensitivity, socioeconomic factors, and geography may heighten climate change’s impacts on the public’s health [20–22].

Infectious disease, pollution, and climate change seem to be connected in different ways. Cardio-respiratory diseases are often attributed to climate change influences [23, 24]. Changing pollen patterns, damp buildings with increased mold exposure, and heat stress are vectors for infection [25, 26]. In response to the higher carbon dioxide levels and warmer temperatures, the number of allergenic plants is increasing, which will cause higher exposure to allergenic pollen [27]. Kenny et al. [28], who assessed the effects of climate change on the cardiovascular system, concluded that excess deaths during heat waves were mainly cardiovascular in origin. The IPCC [29] stressed that, with 2 °C of global warming, extreme heat would exceed critical thresholds for health more frequently by the mid-21st century. Therefore, we expect climate change to continue to increase cardiovascular disease risk [30], with related economic costs for prevention, treatment, and rehabilitation.

In the short- to medium-term, the health impacts of climate change will be determined by populations’ vulnerability and resilience [19], which is also influenced by the state of mental health. “Climate change anxiety”, “ecological grief”, “eco-anxiety”, and “solastalgia” [31–33] are terms that capture the emotional responses to the climate crisis. For example, Reyes et al. [12] define climate change anxiety as “the fear, frustration, and concern over environmental and ecological issues, which stems from the awareness of the increasing life threats from climate change.” Recent scientific evidence shows that climate change, environmental pollution, and pandemics might negatively affect mental health [34–36]. Still, little consideration has been given to how climate change may affect mental health even though the link between extreme anxiety reactions and severe weather disasters (e.g., floods, forest fires, cyclones) was often established [37].

Consequently, the highly mediated adverse consequences and increased awareness underline climate anxiety as a potentially widespread psychological phenomenon [38]. In this context, we considered it relevant to include in the interview the climate change anxiety scale, as a scale for self-perceived climate change anxiety, developed by Clayton and Karazsia [31].

Considered an “agent of metamorphosis” [39], climate change requires changes in human behavior and value systems [40] since human behavior substantially contributes to climate change. Therefore, responding to climate challenges requires understanding people’s perceptions of and attitudes towards climate change risks that are at the core of social resilience that positively influence adaptative behavior [41]. People with different experiences and history perceive climate change differently [42]. Consequently, it was important to reveal how different age cohorts relate to climate change-health nexus.

To respond to our EQ “How do the two selected generations understand and interpret climate change-health nexus?”, we built upon two papers that integrated a qualitative approach to reveal the link between climate change and health: [43, 44]. The five dimensions and the 12 themes were drawn from the indicated studies. The five dimensions were: (i) Views of individual and community health; (ii) Knowledge around climate change; (iii) Perceived health impact; (iv) Attitudes towards climate change; (v) Behaviors related to climate change. Climate anxiety [scale validated by Clayton & Karazsia [31]], considered a defining feature of Generation Z [14], was included in section iii) Perceived health impact. Practically, the climate change-health concern nexus was depicted by illustrating the views, perceptions, knowledge, attitudes, and behavior of the participants, who are valued as “an instrumental dimension in the climate adaptation and mitigation process” [45]. Knowledge can influence people’s attitudes towards climate change and their willingness to act and support mitigation policies [46]. Therefore, it seemed relevant to study what people know about climate change. Masud et al. [47] warned of the importance of revealing how people perceive climate change and the extent to which they were keen to behave in a climate-friendly manner. Since climate change can be a frame within which tangible behaviors (e.g., recycling, diet) can be placed [48], an important step was to identify environmentally relevant behaviors .

#### Selection of the generations

“Generation” is defined as a given cohort group where all members are born in a limited span of consecutive years, of about 20–25 years [49, 50]. Individuals in this group share their age, location, significant life events, behaviors, and beliefs [5, 51]. Two generations were selected for analysis: the Millennials (Gen Y or the Internet generation, as they are often called) and Gen Z (Generation 2020 or iGen, as they are often referred to).

The Millennials cover the period 1980 to 1999 [52, 53], and they came of age during the emergence of reality television, influenced by popular culture [54]. They are considered digital natives whose daily activities are mediated by digital technologies [55, 56]. Millennials are thought to be highly educated in many aspects, with a high ability to access vast amounts of information easily [49, 57]. In addition, they are perceived as environmentally-conscious individuals [52].

Gen Z is those born up to five years before or after 2000, currently aged 16–26 [14]. They are even more hyperconnected and facile with computers, the Internet, and technology than the Millennials [57]. Unlike the previous generations, Gen Z questions the status quo [58]. The New Future of Humanity survey applied to 10,000 18-25-year old people across 22 countries showed that 41% of respondents considered global warming the most important issue humanity faces [59]. Since 2018, activists belonging to Gen Z have been promoting firm public action on climate change, inspiring the next generation – the Alpha activists [14, 60].

The Millennials and the coming generations, including Gen Z, are technologically adept, far beyond the capabilities of their older peers. Also, the Covid-19 pandemic further illuminated the value of technology across all age groups. We learned that being digitally connected could offer solutions to everyday people’s needs and habits [61]. These aspects weighed heavily in selecting these two generations because digitization is often an ideal tool in climate science communication [62].

As Gen Z enters the labor market, in many cases, their first-level supervisors and/or superiors will be the Millennials. Although Millennials and Gen Z are often studied together (because they have many common characteristics) and in opposition to other generations [63], a clash between these two generations in terms of values, attitudes, and behavior, or lack of understanding among the generations [64] about climate change issues may lead to conflicts between these two cohorts.

Consequently, Millennials will likely encounter challenges managing climate change work and communicating with Gen Z. Moreover, Gen Z will surpass the Millennials (by 2030), with more than one-third of the population identifying as Gen Z [65]. Another reason for selecting these two generations is that along with the Millennials, Gen Z will form the majority of the voting-age population across the European Union (EU), and their views and expectations will matter when designing policies [66], including the climate change and health ones. In this context, the European Parliament [66] recommends that the EU policies address Gen Z from a young age as active citizens who need to be protected and empowered. Understanding these generations is very important since they will significantly shape the future climate change strategies landscape.

Because of the inconsistency in the time span for each generation often reported in the literature [67–69], which is more evident in Europe because of the continent’s different historical circumstances [70, 71], any comparison of the results of the present study should be viewed with caution.

To sum up, the concept of “generation” has been central in analyzing and communicating human-induced climate change [72]. As long as climate change is mainly depicted as an intergenerational conflict, effective responses to climate change are closely related to the knowledge of differences and similarities between generations. While most of the research emphasizes differences between younger and older generations, the present study advances the climate change literature by focusing on two young generations, Millennials and Gen Z, because teenagers and young adults are the ones whose lives will be more affected by climate change [73]. The connection between climate change and health can be a valuable way to engage people with the broader issue of climate change [74]. Therefore, it is important to understand how people make these connections to their daily lives. Generational differences in these understandings and perceptions matter—as younger generations like Millennials and Gen Z will probably be more impacted than older generations. Distinguishing between the views of Millennials and Gen Z on climate change and health can provide us with information on where efforts are needed to educate and engage these groups with climate and health. The study offers an intergenerational understanding of similarities and differences in the climate change-health nexus that can reflect potential conflicts and critical points where intervention is needed.

### Methodology

#### The interview and data analysis

The authors opted for a qualitative research design to understand the nature of climate change-health nexus from the perspective of two generations. Qualitative research is considered to better capture the influence of multi-context environments (political, economic, cultural, social) in which climate change perceptions are evolving [75]. A thematic analysis approach was used to analyze the qualitative data that was reported using the *COREQ* checklist [76] (Appendix 2 offers further explanations about the sampling method, setting for data collection, method of data collection, respondent validation of findings, method of recording data, or inclusion of supporting quotations).

The interview questions elicited responses to the five dimensions presented in the previous section. For the climate change anxiety scale, a 5-point Likert scale was used (1 = never, …, 5 = very often). At the end of the interview, participants were asked about their age, education, monthly average income/family, and the existence of chronic diseases.

We interviewed 41 Romanians (20 from Millennials and 21 from Ge Z). At the 20th interview and 21st, respectively, “theoretical saturation” [77] was reached, meaning new meanings could no longer be revealed. The sampling strategy implied selecting participants from different groups (level of education, age, income, and living area – rural and urban). The semi-structured interviews lasted between 30 and 45 min. They were audio-recorded, transcribed verbatim, and coded by thematic analysis using the *Quirkos Analytical Software* program (version 2.4.1).

The interviewer (who was one of the authors) received, besides the core questions, a set of additional questions to guide her within the interview process (“interview guide”). Content validity was addressed by the interview’s questions about all relevant areas of views, values, and perceptions, potentially indicating the climate change-health nexus. Participants were instructed on the aim of the research, and confidentiality and anonymity were ensured.

**Fig. 1:**
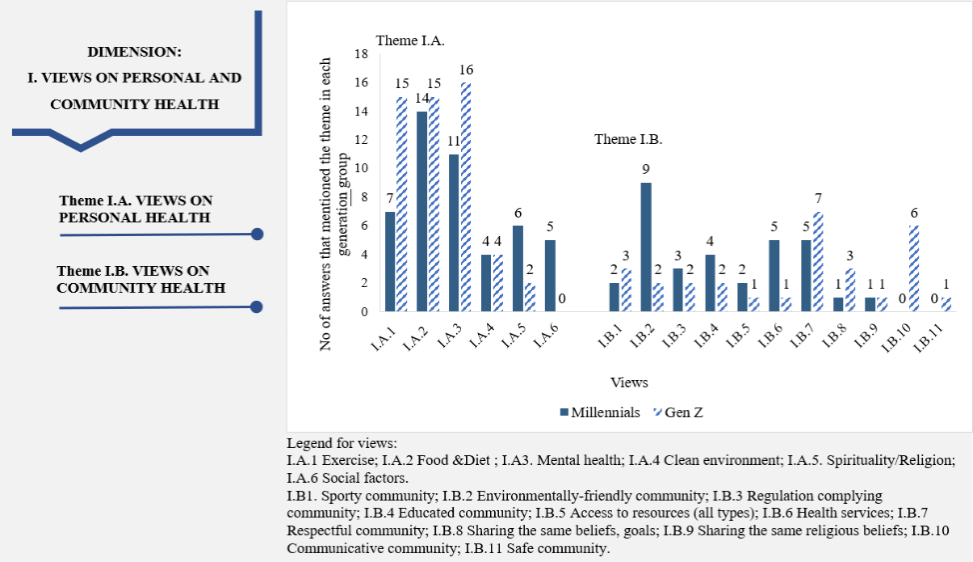
Dimension 1: “Views on personal and community health” in the climate change-health concern nexus (dimension, themes, views, and the participants’ associated numbers of answers)

During data analysis, interview transcriptions were uploaded to the *Quirkos* program, often used in social sciences to perform a thematic analysis. *Quirkos* is a helpful tool for organizing large amounts of textual data. The interview transcripts were read several times to identify, for each of the five sections, the participants’ views (understood as units of information with a commonality of content, “thematic codes” or “Quirks”). The authors agreed on a set of views for each of the 12 themes belonging to the five dimensions. The number assigned to each bullet (Fig. 1A and B, Annex 1) indicates the number of participants’ statements associated with that view. The higher the number of opinions, the bigger the bullet becomes. The participants received the transcripts and were asked to correct any perceived inaccuracies [78]. In the Results section, participants’ perceptions are exemplified with direct quotes [79]. Each quote is identified with the participant study number and a letter (M from Millennials and Z from Gen Z ).

#### Aspects of climate change in Romania

Climate analyses show for Romania a progressive increase in the average air temperature throughout the 21st century, in all seasons, but more pronounced in the summer and winter seasons. The warmest year recorded was 2015. For 2012–2017, the annual thermal deviations were higher than 1.5 °C compared to the multi-year average in 1961–1990 [80]. Almost 13.5 million hectares represent land used for agriculture (57% of the total area of Romania) [81]. The agricultural areas in Romania are affected by frequent drought (around 7 million ha), temporary excess of water (about 4 million ha), water erosion and landslides (about 6.4 million ha), and compaction (approximately 2.8 million ha). Drought is the main limiting factor beacuse it affects most of the agricultural area, which will increase people’s vulnerability [82].

## Results

For the interview, 41 people were selected according to the sampling procedure and interviewed. Other seven individuals were unable to participate due to time constraints (four from Millennials and three from Gen Z). The demographic characteristics of the study population are presented in Table 1. The mean age of the Millennials was 33.71 years and 20.8 years for the Gen Z participants, respectively. The distribution of gender, living area, and presence of chronic diseases were similar between the two generational groups.

**Table 1:** Summary profile of the participants

Variable	Millennials participants (*n = 20*)	Gen Z participants (*n = 21*)
Gender (% out of the sample)	50% female	61% female
	50% male	39% male
Education (completed level)		5% 10 classes
	45% 12 classes	90% 12 classes
	55% university	5% university
Living area (% out of the sample)	50% of urban area	52% urban
	50% of rural area	48% rural
Average age (years)	33.71	20.8
Chronic diseases (% out of the sample)	5%	5%

**Fig. 2:**
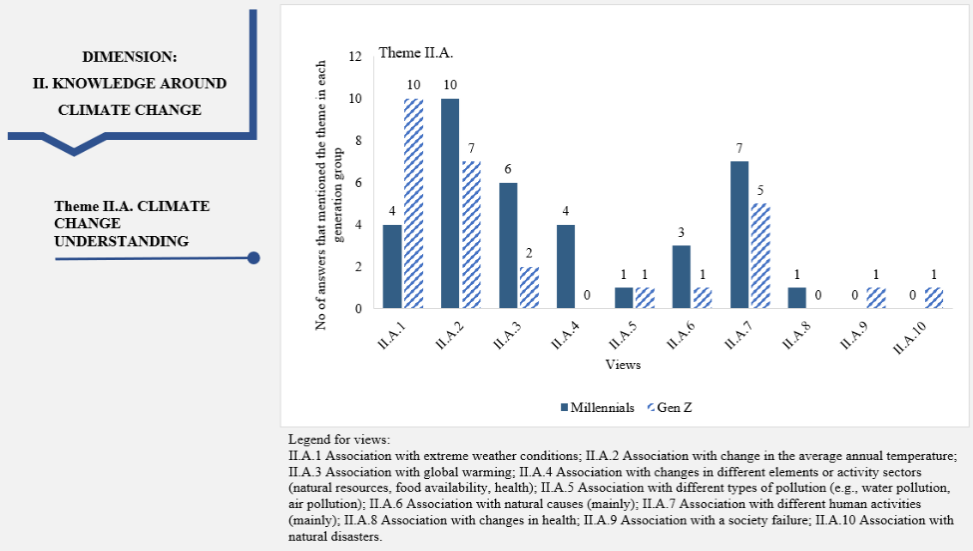
Dimension 2: “Knowledge around climate change” in the climate change-health concern nexus (dimension, theme, views, and the participants’ associated numbers of answers)

**Fig. 3:**
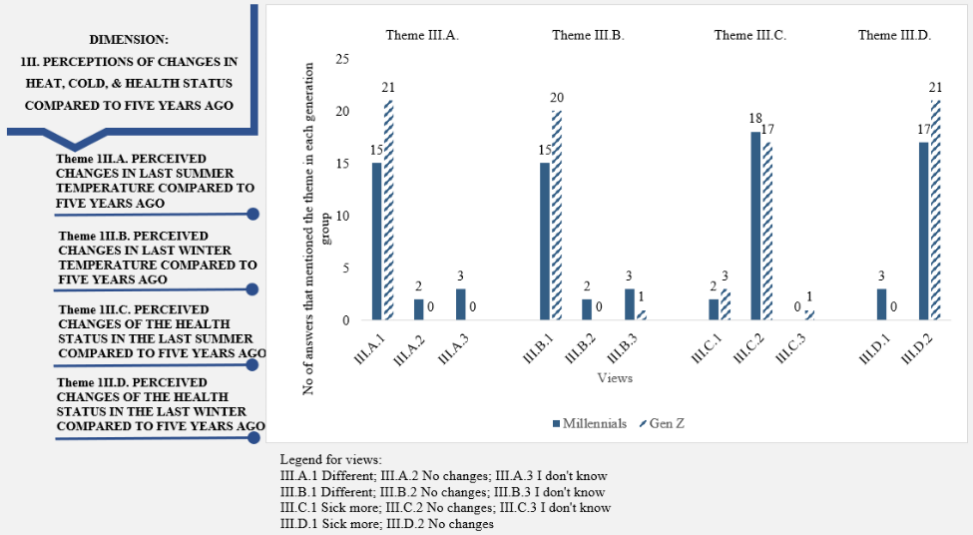
Dimension 3: “Perceptions of changes in heat, cold, & health status compared to five years ago” in the climate change-health concern nexus (dimension, themes, views, and the participants’ associated numbers of answers)

**Fig. 4:**
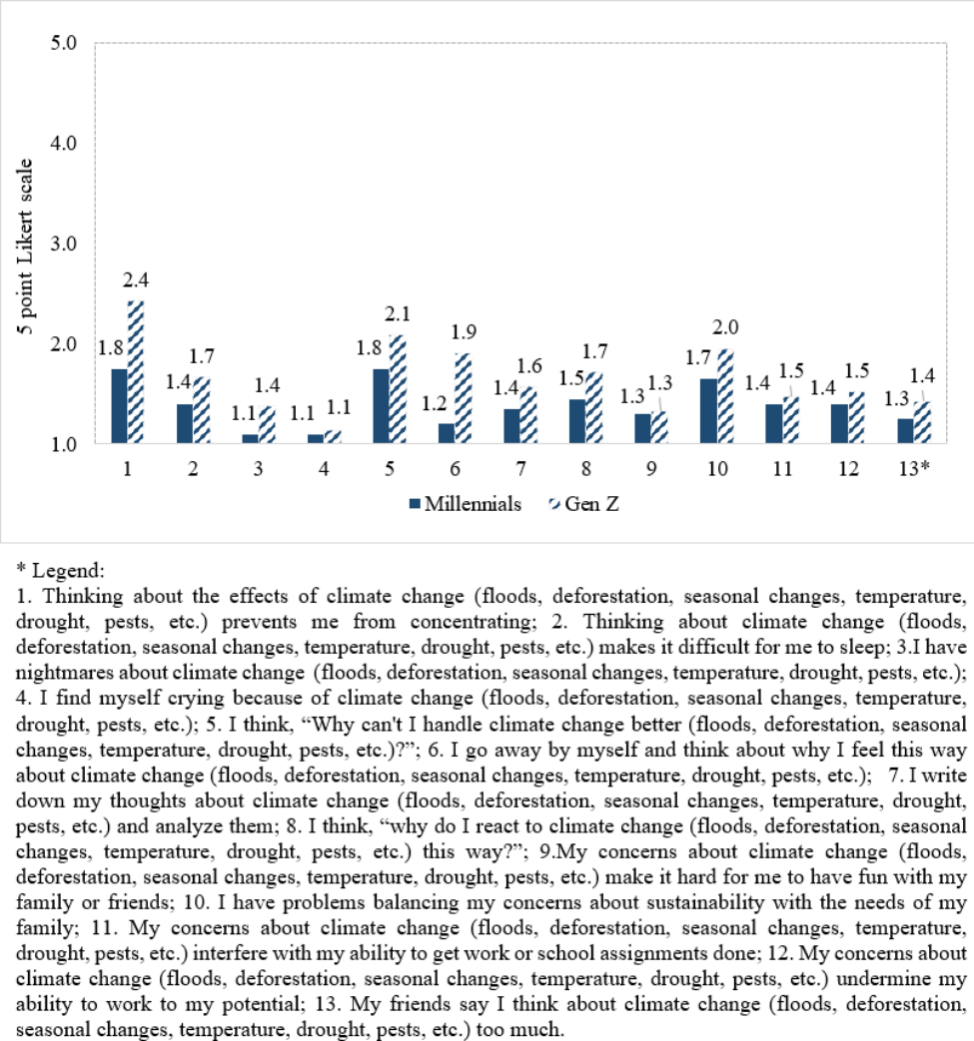
Millennials’ and Z participants’ scores for climate change anxiety (1 = never, …, 5 = very often)

**Fig. 5:**
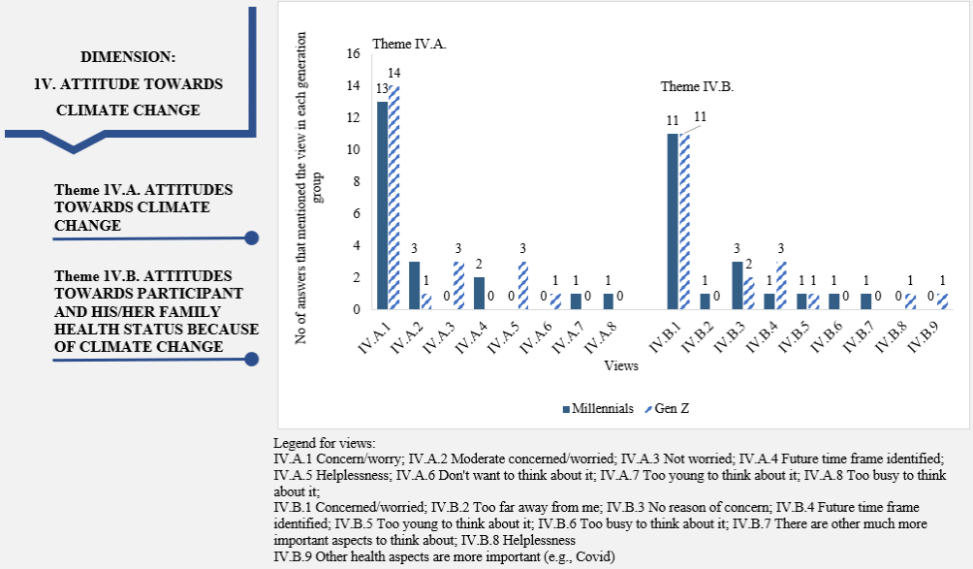
Dimension 4: “Attitudes towards climate change” in the climate change-health concern nexus (dimension, theme, views, and the participants’ associated numbers of answers)

**Fig. 6:**
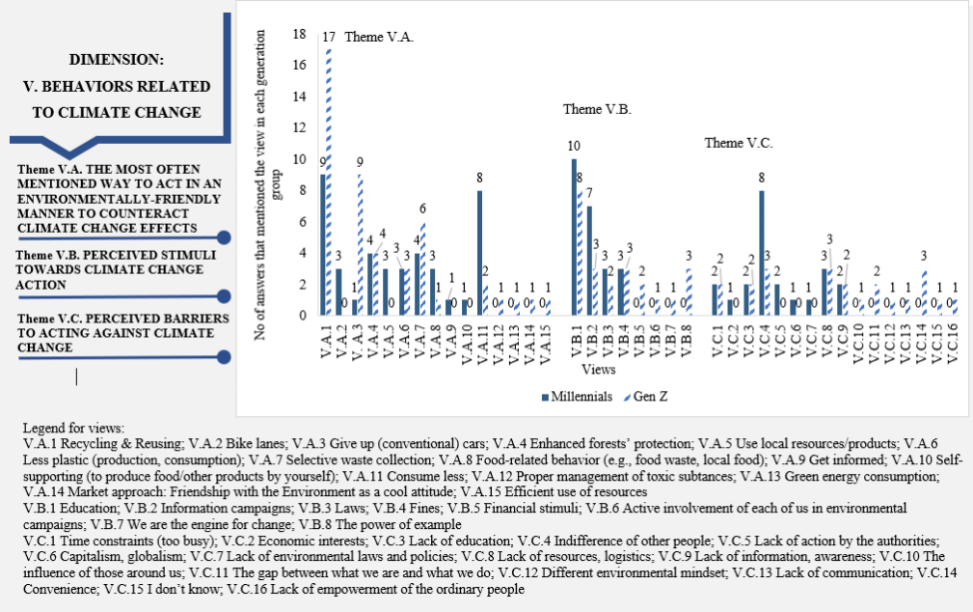
Dimension 5: “Behaviors related to climate change” in the climate change-health concern nexus (dimension, theme, views, and the participants’ associated numbers of answers)

The similarities and differences in how the Millennials and Gen Z participants posited climate change-health concern nexus are presented in Figs. 1, 2, 3, 4, 5 and 6. The matrix of the participants’ views (the “Quirks”) generated in *Quikos* software 2.4.1 is visible in Fig. 1A and 2A (Appendix 1).

“Views on personal and community health” dimension: In the present study, exercise, food and diet, and mental health were the most frequently mentioned drivers of personal health. Around 60% of Millennials and between 35% and 60% of Gen Z participants considered that these aspects made an individual healthy (Fig. 1). However, only 20% (n = 4) of Millennials and Gen Z participants referred to environmental aspects (e.g., clean air) in defining personal health. For example, 3Z voiced that *“(…) the environment is relevant for health. It is imperative because clean air helps the lungs.”* A difference between the selected generations regards the role of social aspects in the maintenance and restoration of personal health, which were mentioned only by the Millennials (25% of them), while Gen Z completely ignored them.

Alternatively, when asked what makes a community healthy, most participants mentioned education and an environmentally friendly community. While 45% (n = 9) of the Millennials identified environmental factors as determinants of community health, only 9,5% (n = 2) shared this opinion (Fig. 1). A participant explained that *“if all the people (…) used less plastic and produced more food in their yard as they did decades ago, it would increase the health of the environment, and thus their health” (3 M).* Other views about what makes a community healthy that were present with a different frequency in each generation were health services (mentioned by none of Millennials and 29% of Gen Z) and communicative community (indicated by 25% of Millennials and 0.5% of Gen Z). The answers where the number of participants was similar in each generation were, for example, about being a sporty community (10% of Millennials and 14% of Gen Z) and regulation-complying community (15% of Millennials and 10% of Gen Z).

“Knowledge around climate change” dimension: Most views expressed within this dimension can be found in both generations, but with a different frequency. About 47% (n = 10) of the Z people related the phenomenon of climate change with extreme weather conditions compared to only 20% (n = 4) of the Millennials (Fig. 2). The percentage of the latter increased (50% of the Millennials, n = 10) when it came to changes in the average annual temperature that they thought of in connection with climate change. Comparatively, only 33% (n = 7) of Gen Z shared this view. It can be inferred that the participants had difficulties distinguishing climate change from the weather. For example, climate change was understood as “*severe extreme weather phenomena, such as floods and droughts” (18Z).* Furthermore, both generations tended to associate climate change mainly with anthropogenic causes rather than with natural ones: *“Climate change is mainly due to anthropogenic activity” (7 M)*, “*(…) first of all, the burning of fossil fuels such as natural gas, coal, or oil are those that produce such changes that can sometimes be catastrophic and, why not, irreversible” (6Z)*. One representant of each generation associated climate change with different types of pollution (Fig. 2).

“Perceptions of changes in heat, cold, and health status because of climate change” dimension: Most participants perceived changes in last summer and winter temperature compared to five years ago. However, the spread of this perception in each generation is different. Thus, 75% (n = 15) of Millennials and 100% (n = 21) of Z people voiced that they observed differences in last summer temperature compared to five years ago (Fig. 3). In addition, they mentioned warmer temperatures in winter and a lack of snow. They referred to both the increasing number of storms and the prolonged periods of drought in summer: *“If before, excessive heat was associated with precise geographical locations (seaside, plains), now the heatwave occurs even in mountainous areas” (15 M); “The winters have warmed up, we have less snow, less frost” (4Z).* Both generations shared similar perceptions regarding their health status in the last summer compared to five years ago, with most participants from each generation signaling no change (90% of Millennials and 81% of Gen Z). A small difference appeared in their view about health status in the last winter compared to five years ago, with 85% (n = 17) of Millennials indicating no changes and all Gen Z participants mentioning no changes.

We observed that likewise the perceived impact of climate change on physical health (Fig. 3, Themes III.C and III.D), participants from both generations assigned low scores for all 13 items of the climate change anxiety scale (Fig. 4). “Thinking about the effects of climate change (floods, deforestation, seasonal changes, temperature, drought, pests, etc.) prevents me from concentrating” received the highest score from both Millennials and Gen Z: 2.4 points and 1.8 points, respectively.

“Attitudes towards climate change“ dimension: The views of both generations were similar within this dimension for both themes (Fig. 5). Comparing the themes, a larger number of Millennials and Gen Z stated that they were more concerned about climate change than those worried about climate change’s impact on their health (Fig. 5). Several responses included the reference to age and the impact of climate change on health as a future phenomenon: *“(…) I think things could worsen in the near future, that is, we will have very high temperatures that could worsen chronic diseases of family members” (15 M); “No, not yet. I’m too young” (1Z); “I am not too worried about my family’s health being affected by climate change. Maybe I don’t see how this could affect my family’s health, so I don’t worry” (11 M).*

“Behaviors related to climate change” dimension: The most mentioned way to behave in an environmentally friendly manner to counteract the effects of climate change was “Recycling/Reusing” (Fig. 6). This is a common opinion of both Millennials and Gen Z participants: *“I recycle and use reusable products to reduce waste” (21Z).* However, differences in how common this view is within generations should be highlighted: around half of Millennials compared to Gen Z (45% of Millennials vs. 81% of Gen Z). Another difference is observed for giving up (conventional) cars (5% of Millennials vs. 43% of Gen Z) and consuming less (40% of Millennials vs. 0.5% of Gen Z). “Selective waste collection”, “Consume less”, and “Enhanced forests’ protection” were other behaviors mentioned by the participants: *“Illegal deforestation should be stopped and sanctioned to prevent most floods” (17Z).*

In general, when Millennials and Gen Z participants were asked about stimuli and barriers to action on climate change, there was a tendency visible in both generations to dissociate themselves from responsibility for tackling climate change (Theme V.B, Fig. 6). Only one Gen Z interviewee expressed that “we are the engine of change”, when referring to the stimuli towards action: *“We are the change. We need to be an example to ourselves and our friends, families, and even strangers” (6Z).* However, both generations acknowledged peoples’ behavior and attitude as barriers to acting against climate change. They often mentioned the “indifference of other people” as a barrier: *“The barriers are primarily the laziness and indifference of individuals” (3Z)*. A difference between generations regarding perceived barriers is that Gen Z mentioned a wider variety of barriers compared to Millennials.

## Discussion and final remarks

The present study reflects a contextual understanding of the climate change-health nexus of Millennials and Gen Z participants, which is needed to become aware of the dynamics across generations. Individuals bring their perceptions, attitudes, and behaviors about climate change to their group connections, and, in turn, the generational group affiliations influence climate change individual attitudes [83]. Thus, the importance of an intergenerational understanding of the climate change-heath nexus is evident. Five dimensions of climate change-health nexus with the extracted views were qualitatively explored.

The findings revealed a set of commonalities and differences in understanding the climate change-health nexus between the participants representative of each of the generations examined. For “Views of personal and community health”, mainly physical and mental health was brought to the fore. At the same time, the references to a clean and balanced environment were surprisingly less mentioned by only one-quarter of participants from each generation. Aspects of physical health (food, diet, sport) and mental life were frequently stated when people expressed their opinion about the meaning of personal health (Fig. 1). These results are in line with those reported by Cardwell and Elliott [43] for citizens from Southern Ontario, USA. From a practical perspective, education and information campaigns that bring to the fore the interplay between climate change, environment, and human health should consider mainly Gen Z because it is a generation not fully formed [67], which allows easier modeling of perceptions and behaviors. A difference between generations is illustrated by the fact that only the Millennials mentioned that social factors influenced personal health. Participants defined the social factors as interactions that are useful and enjoyable. This view implies a deeper connection of Millennials with their peers compared to Gen Z, who are more self-centered about their health. The “social connections” mindset of the Millennials is visible in their definition of a healthy community, too. They mentioned that a healthy community should be environmentally-friendly, regulation-complying, and respectful, all requiring cooperation between people. When defining a healthy community, Gen Z also perceived the social side of it, considering that a healthy community is communicative and respectful. The existence of these beliefs suggests that a program aiming to improve community health and focusing on these generations should highlight the social component of a healthy community as a motivating factor. However, messages should have a distinct focus for each generation, depending on the specific aspects relevant to each of them. Thus, for example, if a program aims to stimulate healthy behavior within a community, such as the adoption of a healthier diet, engaging Gen Z in an activity that requires communication (e.g., exchanging views, writing reviews) can work better than highlighting the environmental benefits associated with the consumption of the healthy foods. The latter approach may be better received by Millennials, who could be more prone to adopt a healthy diet if, for example, the foods’ lower water and carbon footprint are promoted.

For the “Knowledge around climate change” dimension, we found a tendency of participants to define climate change mainly as extreme weather events such as floods, heavier rainfall, and higher temperatures during winter (Fig. 2). These views were probably shaped by their interaction with the environment over the years. We are aware that climate change is invisible to ordinary people, as climate change relies on statistical data compiled over long periods [84]. Similarly, Weber [85] warns that climate change is not easily detected by personal experience. People often falsely attribute events to climate change and fail to detect changes in climate, which indicates confusion between climate variability and climate change (for example, one unusually cold year followed by an unusually warm year are not signs of climate change). Similar perceptions of participants from developed and underdeveloped countries were reported in the literature on climate change [86, 87].

The fact that both generations recognize certain characteristics of climate change within the “Knowledge around climate change” dimension proves that a knowledge foundation exists in both cases. This can be used to create and enhance environmentally friendly behaviors, such as using green energy. The number of answers associated with these views differs between the interviewed generational participants. Gen Z people associate climate change more with extreme weather conditions than Millennials. Following this difference, we can suggest that intervention measures must be finetuned to each generation’s most frequently recognized aspects. A message to Millennials should associate climate change with the change in the average annual temperature, while for Gen Z, it should highlight extreme weather conditions. The association of climate change with extreme weather conditions is an encouraging finding because various authors suggested higher engagement with climate change and pro-environmental behaviors when people connect climate change with their experience of extreme weather [88]. A greater number of media news items that bring to the public’s attention extreme weather events can have the merit of contributing to the increased climate change actions. Previous qualitative research [89] highlighted that Romanian participants perceived climate change as mainly a human-induced phenomenon. Figure 2 shows that more Millennials associated climate change with global warming than the Z’s representatives. Stokes et al. [63] found that Millennials and Gen Z were more convinced of anthropogenic climate change than older generations. Other research identified that younger generations perceived the seriousness of climate change more than older respondents, who were more skeptical and less concerned about climate change [90, 91]. As Cook et al. [92] and Leiserowitz [45] pointed out, there is a broad public perception that climate scientists contradict over the fundamental cause of global warming, which influences what people think about it.

For the “Perceptions of changes in heat, cold, and health status compared to five years ago” dimension, we revealed that while most interviewees perceived changes in summer and winter temperature, they failed to articulate how climate change affected their health status (Fig. 3). Few of them said they were sicker because of the higher number of colds, headaches caused by higher temperatures, and more severe allergy symptoms. One explanation could also stand in perceiving climate change as a long-term problem, and many young generations have not yet experienced the irreversible changes.

This finding is in line with the relatively low number of respondents from twenty-four countries who were not very much concerned about the impact of climate change on their health (14% of the total of 1,100 individuals) [93]. Akerlof et al. [94] believed that there was little research on public perception of the human health impacts and risks associated with climate change. The need to become more aware of the connections between climate change and health is justified by the scientific evidence [95] that has shown that the impact of climate change has immediate and long-term indirect effects on public health. A study by Haq et al. [93] suggest that a warmer climate may mostly affect those suffering from cardiac diseases. In comparison, colder weather may cause an increase in the prevalence of coughs/colds, headaches, or asthma. Concern about the climate change-health nexus is worthy of investigation because it can predict the willingness to change climate-related behavior [96]. That is why the lack of understanding of climate change as a health risk for ordinary people represents a significant barrier to behavior change [43]. An effective way to make people aware of the climate change impact on their health is to reframe climate change understanding more as a health issue than an environmental one. When climate change is described as a human health issue, a larger audience finds the information useful [97], which can change perceptions and attitudes.

Similarly, Myers et al. [74] found that framing climate change as a public health risk elicited emotional reactions that could support climate change mitigation and adaptation. Consequently, the extent to which the participants are aware of the health relevance of climate change remains unclear. This lack of clarity highlights the need to inform and educate people about the health risk associated with climate change, which can become an essential function of educational and public health systems.

Chen et al. (2020) observed that greater exposure to climate change is intuitively associated with higher health symptoms, including psychological ones [98]. Xu et al. [99] evidenced the increased negative impact of higher temperatures on childhood mental health due to reduced participation in physical activities. In the present study, the anxiety scores for Gen Z were slightly above those of the Millennials, suggesting a higher emotional impact of climate change for the representatives of the younger generation (Fig. 4). This is consistent with other research that showed that younger generations are more concerned than older generations about climate change. The young generations will experience more of the worst impacts because they will live longer in the future [13]. Climate change was indicated by American Psychological Association [100] *apud* [13] as the most significant source of stress for Gen Z than for older generations. In a study trying to understand feelings and thoughts associated with climate change among young people in ten countries, it was shown that distress is present both in countries where the direct impacts are less severe (e.g., the UK) and in countries that are experiencing extensive physical impacts of climate change (e.g., Philippines) [101]. However, the percentage of those declared extremely and very worried was higher in the Philippines (84%) and India (68%) than in the UK (49%) and France (58%) [101]. Based on findings reported in climate change anxiety literature, climate change anxiety does not necessarily predict greater uptake of self-initiated efforts to reduce the harmful effects of climate change [77, 102] because anxiety may draw out avoidant thinking and behaviors [102]. Despite participants’ low climate change anxiety, they offered rich and documented solutions of how they fight climate change (e.g., forest protection, less use of plastic products, recycling and reusing behaviors, which are illustrated in the fifth dimension “v) Behaviors related to climate change”).

For the dimension “Attitudes towards climate change”, a similar pattern of views is visible for both generations. Most participants stated they are concerned about climate change, but only half of them about the impact on their health. Participants’ answers showed that the concern for climate change surpasses the concern for climate change impacts on health (Fig. 5). An explanation can be found in the climate change literature. Fischhoff et al. [103] consider that personal exposure to adverse consequences increases perceptions of risk translated into great concern, and familiarity with a risk acquired by exposure without negative consequences can also lower perceptions of its riskiness.

Similarly, Weber [85] suggests that if people perceive climate change as a gradual change from current to future values on several variables (e.g., precipitations, average temperatures), the risks posed by climate change would appear familiar and, to some extent, controllable. Another possible explanation is that we interviewed young participants with self-reported good health status (only 5% of the interviewees of both generations reported chronic diseases). Many participants tended to link health risks with a future time frame or an older age. This is why we can assume that they consider climate change risks mainly in the future, which makes them override possible consequences, like those for their health.

The relatively low concern for climate change impact on health is in line with their previous responses about the recent perceived changes in health status (Fig. 3, Themes III.C and III.D). The polarized perception about, on the one hand, the increased perception of temperature changes in winter and summer (Fig. 3) and, on the other hand, the near absence of a perception of a change in health status due to these changes (Fig. 3), make us conclude that Romanian Millennials and Gen Z people do not perceive a nexus between climate change and their health condition. This suggests that intervention points to increase awareness of climate change effects on health should be considered because several people said they were too young, too busy, or there were other much more critical health issues to think about.

An environmentally-friendly behavior is the main aim of mitigating climate change [42]. The findings underscored an encouraging appreciation for the “Behaviors related to climate” dimension. The selected participants could be considered knowledgeable about environmentally friendly behaviors that could counteract climate change, stimuli, and barriers to climate change mitigation actions. Recycling was one of the most mentioned ways to act environmentally friendly by the sample as a whole (Fig. 6). This is not surprising since recycling is one of the most used strategies to combat climate change, with significant benefits for adaptation to climate change and practical mitigation [104, 105]. While both generations mentioned a wide variety of behaviors, there are differences in their frequency within each generation (Fig. 6). Recycling and reusing may be successfully proposed for both generations, but it was mentioned twice more often by Gen Z participants. In addition, Millennials may be more receptive to actions focused on lowering consumption. At the same time, the younger Gen Z may be more prone to give up (conventional) cars and selectively collect waste in efforts to fight climate change (as these were the most frequently mentioned behaviors by Millennials and Gen Z people, respectively).

Regarding stimuli and barriers to fight climate change, similar views in both generations highlight a common thinking pattern. This can be used in marketing actions to strengthen the feeling of belonging to the same community of both generations, increasing their solidarity, cooperation, and engagement in climate-friendly behaviors. Gen Z indicated a higher variety of stimuli and barriers, implying that they can be better informed or more willing to communicate what they know about climate change. While it is evident that interviewed people had many opinions about what changes were necessary to counteract climate change impacts, when asked about stimuli and barriers to acting, most of the respondents tended to place the involvement and, practically, the responsibility of acting on others. Thus, the participants can be depicted rather as observers than players since they do not tend to see themselves (their behavior, their contribution) as playing a part in fighting climate change. An explanation could be that respondents do not understand how they can contribute to fight climate change or the importance of individual actions within the collective effort. Both generations undergo what Stephen Gardiner [1] called “intergenerational buck-passing” meaning that each generation does little to fight climate change and passes the problem to the next generation, amplifying the climate crisis over time [106].

Consequently, it is essential to inform and educate the young generations about the relevance of their climate-friendly activities. Adapting to climate change requires collective action, and understanding the factors predicting pro-environmental behaviors is essential. The investigation of Gen Z behavior that can contribute to fight climate change is relevant because, in the next 30 years, this will be the generation that will decide upon the critical actions to be taken to solve the climate change issue [42]. Similarly, in a qualitative interview in five European countries, people had clear views on the “right” behavior, but their actions were sometimes exempted [107]. Considering that climate change adaptation and mitigation are in line with the terms “responsibility” and “action” [108], this finding raises concerns, and further investigation is required in climate change research.

We acknowledge several limitations of the present research. One is related to the method used for analysis. Since we conducted qualitative research, the findings must be received in the context of their subjectivity. However, it is important to note that data saturation did occur. A qualitative approach also has advantages, such as offering a more nuanced understanding of differences and similarities between the selected groups. Moreover, insights into small-scale studies are better captured by focusing on a small and particular subset of larger audience segments [109]. Additionally, future studies could implement other methodologies to allow generalizability. For example, since 20% of the Millennials associated spirituality/religion with personal health, future studies could investigate the role of religion in modeling climate change perceptions. This is even more important since various papers [110] revealed that cultural beliefs promoted by religion could cause maladaptation. Furthermore, in terms of climate change anxiety, more research is needed to understand the emotional reactions to climate change and investigate whether mental well-being is particularly threatened by climate change.

Finally, regarding the intergenerational reading of climate change, the main differences rely on the number of answers assigned to a view and the existence of different views from one generation to another. For example, we observed many Millennials associating a healthy community with an environmentally friendly one compared to Gen Z participants. At the same time, Gen Z emphasized communication between the community members. However, there is no relevant difference between Millennials and Gen Z participants regarding the perceptions, attitudes, knowledge, and behavior of the five studied dimensions. One important conclusion drawn from the thematic analysis is that the commonalities of views outweigh the differences between the two generations. This is probably because, as Swim et al. [13] said, Millennials and Gen Z share a unique cultural milieu, being both young generations.

## Electronic supplementary material

Below is the link to the electronic supplementary material.

Appendix 2. COREQ checklist additional information

Appendix 1

## Data Availability

The data that support the findings of this study are available here: Petrescu-Mag, Ruxandra Malina (2022), “Climate change perceptions_Health_Generations”, Mendeley Data, V1, doi: 10.17632/h85mzjcxtb.1.
