# Supplementary material for: An intergenerational reading of climate change-health concern nexus: a qualitative study of the Millennials’ and Gen Z participants’ perceptions
Source: BMC Public Health. 2023 Mar 13;23:484. doi: 10.1186/s12889-023-15353-z (PMC10010654; doi:10.1186/s12889-023-15353-z)
Supplement: Supplementary file 1 — Appendix 2. COREQ checklist additional information [file 12889_2023_15353_MOESM1_ESM.docx]

Appendix 2. COREQ checklist additional information

**Domain 1: Research team and reﬂexivity**

*Personal characteristics:* The interviewer was one of the authors (AT). She is a Ph.D. senior researcher at Babes-Bolyai University, Cluj-Napoca, Romania. She previously participated in other qualitative research and focus groups. Her experience in qualitative research ensured no unintended influence on what the subject answered.

*Relationship with participants:* Several participants (four) were already known to the facilitator due to previous common projects. Participants were explained the aim of the research, why they were selected, and what would happen with the interview data. Confidentiality and anonymity were ensured. No financial reward was given for participation.

**Domain 2: Study design**

*Theoretical framework:* A thematic analysis approach was used to analyze the qualitative data.

*Participant selection:* The sampling strategy implied selecting participants from different socio-demographic groups (considering age, level of education, income, and living area – rural and urban). We started from an initial group (people with whom the authors interacted/worked and who were approached by phone) for the recruitment of participants. Then, following the snowballing technique (meaning that a participant recommended another one who was further selected if she/he met the pre-established selection criteria), the final number of participants was reached. 41 people (20 from Millennials and 21 from Generation Z) participated in the interview. 7 persons (4 from Millennials and 3 from Generation Z) refused to participate in the interview because of time constraints.

*Setting:* Data was collected in various settings, depending on the participants’ availability, such as a coffee shop, home of the interviewee, canteen of the interviewee's workplace, and a food court from a mall. The interview was conducted without the presence of other persons. The demographic characteristics of the sample are included in the main text, Table 1.

*Data collection:* The interview guide was provided by the authors. The questionnaire was piloted on five people (before being used in the field) to test the proposed study design and process, and thus to assess the efficacy of the research instrument. The semi-structured interviews lasted between 30 and 45 minutes. They were audio-recorded, transcribed verbatim, and coded by thematic analysis using the Quirkos Analytical Software program (version 2.4.1). Memos were made as soon as possible after each interview to record nonverbal observations and reflections on participant’ responses. At the 20^th^ interview and 21^st^, respectively, “theoretical saturation” was reached, i.e., new meanings could no longer be revealed. The interviewer received a set of questions (“interview guide”) to guide her within the interview process. This “interview guide” comprised core questions and many associated questions related to the central question. Follow-up questions were asked when appropriate.

**Domain 3: analysis and ﬁndings**

*Data analysis:* The participants were provided the transcripts, and they were asked to correct any perceived inaccuracies. Interview transcriptions were uploaded to the Quirkos program by the first author (RMPM) and the thematic analysis was performed by the first and last author (RMPM, DCP). The interview transcripts were read several times to identify, for each of the five dimensions, the participants’ views (understood as units of information with a commonality of content, “thematic codes” or “Quirks”). The authors agreed on a set of views for each of the 12 themes belonging to the five dimensions. While the five dimensions and the 12 themes were built on the presented theoretical framework, before performing the interview, the participants’ views for each of the 12 themes were derived from the data. The matrix of the recurring views, generated in Quikos software 2.4.1, was included in Appendix 1.

*Reporting:* Participants’ quotations were included in Results section, and they aimed to illustrate the ﬁndings. There was consistency between the data presented and the ﬁndings. The 12 themes were clearly presented in the Results section. Additionally, besides participants’ commonalities of views for the 12 themes, we illustrated other participants’ views. To preserve participants anonymity, the participants were assigned a number and letter M (for Millennials) or Z (for Z generation).
