# Supplementary figures and images for: An intergenerational reading of climate change-health concern nexus: a qualitative study of the Millennials’ and Gen Z participants’ perceptions
Source: BMC Public Health. 2023 Mar 13;23:484. doi: 10.1186/s12889-023-15353-z (PMC10010654; doi:10.1186/s12889-023-15353-z)

**Appendix 1**

Figure 1A Millennials’ views


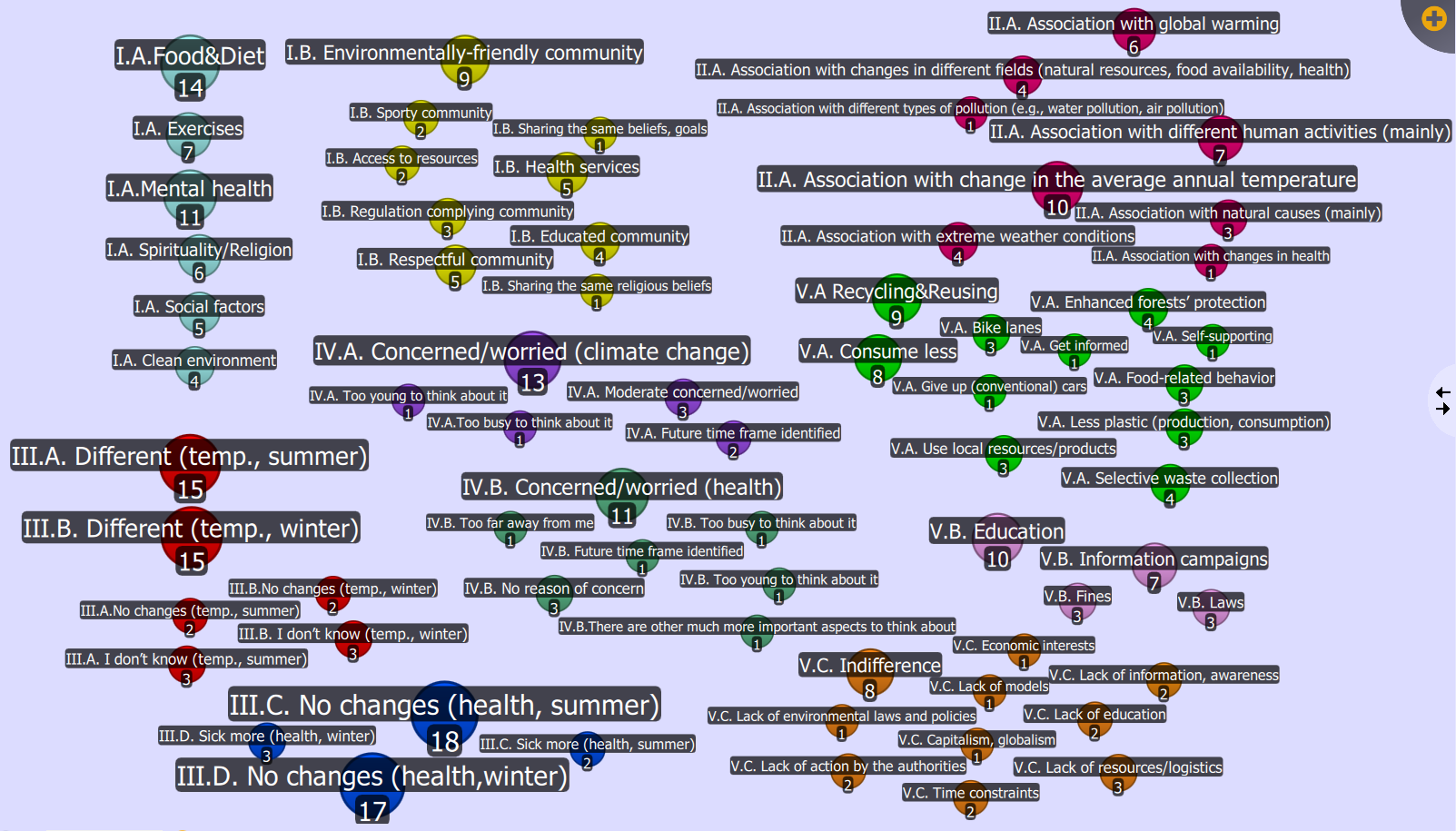


Figure 2A Gen Z’s views


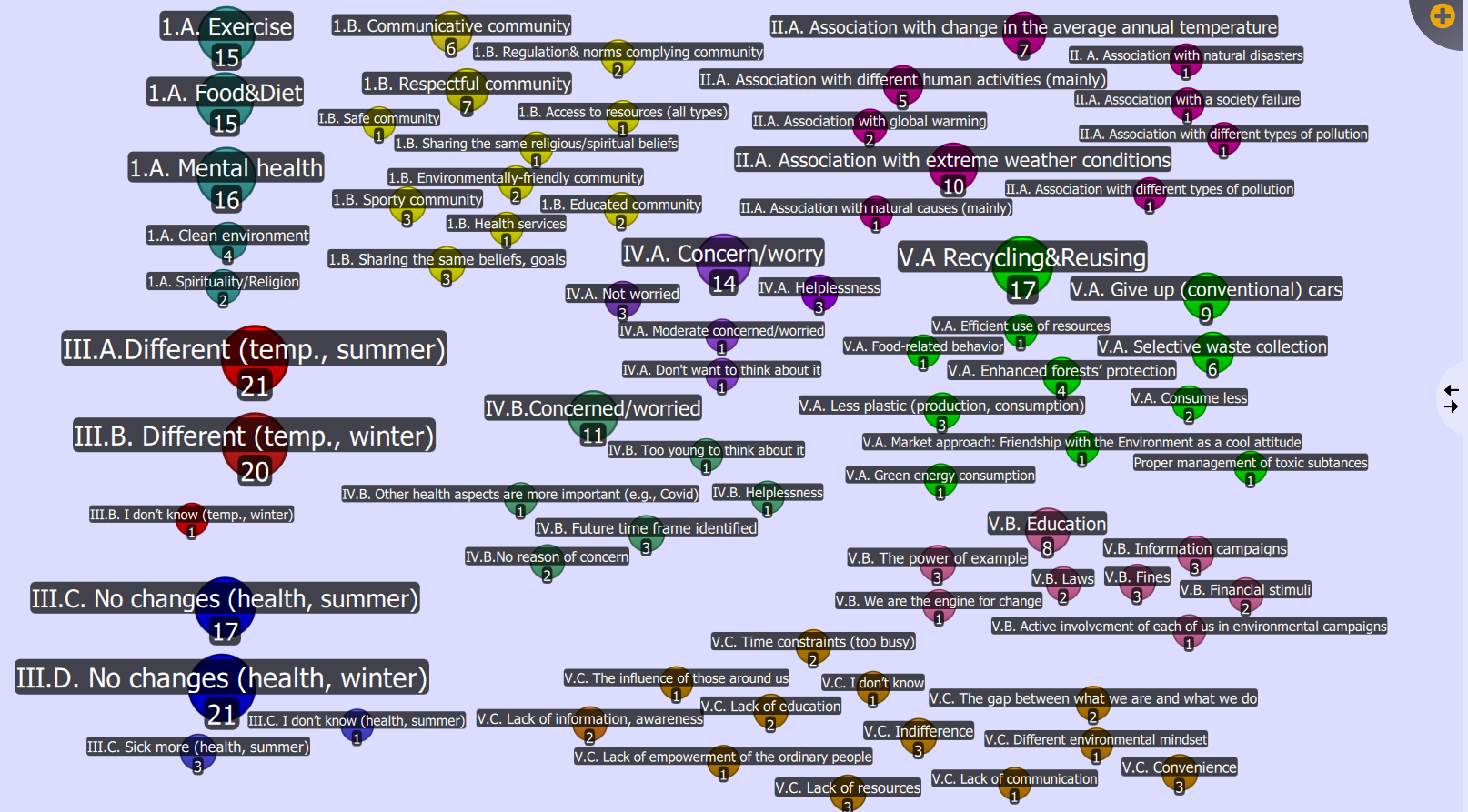

Supplement: Supplementary file 2 — Appendix 1 [file 12889_2023_15353_MOESM2_ESM.docx]
